# Sympathetic Activation and Sleep-Related Movements: Integrating Autonomic, Dopaminergic, and Iron Deficiency Mechanisms

**DOI:** 10.3390/brainsci16050539

**Published:** 2026-05-20

**Authors:** Gulcin Benbir Senel, Lourdes M. DelRosso

**Affiliations:** 1 Sleep and Disorders Unit, Department of Neurology, Cerrahpasa Faculty of Medicine, Istanbul University-Cerrahpasa, Istanbul 34320, Turkey; drgulcinbenbir@yahoo.com; 2 Department of Medicine, University of California San Francisco, Fresno, CA 93701, USA

**Keywords:** sympathetic activation, sleep-related movement disorders, restless legs syndrome, autonomic dysfunction, iron deficiency, heart rate variability

## Abstract

**Highlights:**

**What are the main findings?**

- Sympathetic nervous system activation frequently precedes or accompanies sleep-related motor events, supporting a potential causal role rather than a purely reactive response.
- Iron deficiency and dopaminergic dysfunction interact with autonomic dysregulation, forming an integrated pathophysiological model for RLS, PLMD, and RSD.

**What are the implications of the main findings?**

- Sleep-related movement disorders should be reconceptualized using a multidimensional framework that includes autonomic dysfunction alongside traditional dopaminergic and iron-based models.
- Targeting sympathetic activation (e.g., via CPAP optimization, pharmacologic modulation, and behavioral interventions) may improve both motor symptoms and overall sleep quality.

**Abstract:**

Objective: Recent research has expanded the understanding of the potential role of sympathetic nervous system activation in the implications of sleep-related movement disorders, particularly in the mechanisms of dopaminergic dysfunction and iron deficiency. This multifactorial perspective aims to provide insights into disease mechanisms and opportunities for targeted interventions that address both neurological and autonomic contributors to sleep-related movements. Methods: To synthesize the current evidence on the role of sympathetic activation in sleep-related movement disorders, we conducted a review of the literature to identify studies exploring the intersection of autonomic nervous system activity and motor phenomena during sleep. Results: Studies indicate that sympathetic activation may contribute directly to the initiation and propagation of motor events during sleep. Evidence from electrophysiological studies and heart rate variability analyses in patients with sleep-related movement disorders shows that sympathetic bursts often precede or coincide with leg movements and arousals, suggesting a causal rather than reactive role. Moreover, iron deficiency appears to exacerbate both dopaminergic and autonomic dysfunction, providing a unifying mechanism that bridges these pathways. Conclusions: These findings support a shift from viewing sympathetic activity as a secondary response to arousal to recognizing it as a possible primary trigger of sleep-related motor events.

## 1. Introduction

Recognition of sympathetic nervous system involvement in sleep-related movement disorders is reshaping clinical understanding and management of conditions like Restless Legs Syndrome (RLS), Periodic Limb Movement Disorder (PLMD), and Restless Sleep Disorder (RSD). Traditionally attributed to dopaminergic dysfunction and iron deficiency, these disorders also demonstrate measurable autonomic dysregulation, with heart rate variability (HRV), skin sympathetic nerve activity (SKNA), and other autonomic markers indicating heightened sympathetic tone during sleep. Clinically, patients with moderate to severe RLS and PLMD often present with disrupted sleep architecture, frequent arousals, and elevated cardiovascular risk, patterns now better understood through autonomic assessment. Tools such as HRV analysis, simultaneous noninvasive recording of electrocardiogram and skin sympathetic nerve activity (neuECG), and peripheral arterial tonometry offer practical, noninvasive ways to assess sympathetic activity and may enhance diagnostic precision. Importantly, this perspective informs therapeutic choices: iron supplementation remains foundational in RLS and RSD, but managing sympathetic activation, through optimized CPAP in OSA, use of alpha-2-delta ligands, or lifestyle interventions like exercise and relaxation strategies, can improve both motor symptoms and sleep quality. Understanding the dynamic between arousal mechanisms and movement generation enables clinicians to adopt a more integrative, individualized approach. This evolving paradigm encourages consideration of autonomic tone as both a contributor to symptoms and a target for therapeutic intervention.

Normal sleep is organized into recurring cycles of non-rapid eye movement (NREM) and rapid eye movement (REM) sleep, each characterized by distinct physiological and neurobiological features. NREM sleep progresses from lighter stages to deeper restorative sleep and is generally associated with reduced sympathetic tone, slower heart rate, and relative physiologic stability. In contrast, REM sleep is marked by vivid dreaming, skeletal muscle atonia, greater variability in heart rate and respiration, and intermittent surges in autonomic activity. Disruption of this normal sleep architecture may contribute to fragmented sleep, daytime impairment, and cardiometabolic consequences. Common sleep disorders such as Obstructive Sleep Apnea further destabilize these physiologic patterns through recurrent upper airway obstruction, intermittent hypoxemia, repeated arousals, and sympathetic activation. Understanding the interaction between normal sleep-stage physiology and disorders that alter autonomic balance provides an important framework for interpreting the pathophysiology of sleep-related movement disorders.

Sleep-related movement disorders are increasingly recognized as contributors to sleep fragmentation, daytime impairment, and long-term cardiovascular risk [1,2,3,4]. Traditionally, the pathophysiology of these disorders has been linked to dopaminergic dysfunction and iron deficiency, particularly affecting subcortical and spinal pathways involved in motor control [5]. However, a growing body of evidence points to an additional, perhaps synergistic, mechanism involving the autonomic nervous system, specifically sympathetic nervous system hyperactivity [4,6].

Physiological recordings and neuroimaging studies have demonstrated that sympathetic surges during sleep often occur in close temporal proximity to leg movements and arousals [7]. In some cases, autonomic activation precedes motor activity, suggesting a causal role rather than a secondary response [7]. Moreover, disruptions in sleep stage transitions, particularly in NREM sleep, where leg movements are most prevalent, are associated with increased heart rate variability (HRV) and skin sympathetic nerve activity (SKNA) [1]. These findings indicate a shared neurobiological substrate in which motor and autonomic circuits are co-regulated by brainstem and hypothalamic nuclei.

Recent studies have further elucidated this overlap, identifying specific brain regions, such as the rVMM and preoptic hypothalamus, as sites of convergence for somatomotor and sympathetic output [8]. Meanwhile, iron deficiency, long known to affect dopamine synthesis, may also potentiate sympathetic activity, creating a bidirectional feedback loop that exacerbates sleep-related movements [9]. This integrated model offers a more comprehensive framework for understanding sleep-related motor phenomena, opening new avenues for diagnosis and treatment that target both neuromodulatory and autonomic pathways.

This narrative review synthesizes current evidence regarding sympathetic and noradrenergic activation in sleep-related movement disorders, with emphasis on Restless Legs Syndrome (RLS), Periodic Limb Movement Disorder (PLMD), Restless Sleep Disorder (RSD), and related autonomic phenomena. We integrate findings from human physiological studies, clinical observations, and relevant translational research to propose a multidimensional pathophysiological framework involving autonomic, dopaminergic, and iron-related mechanisms.

## 2. Neurobiological Basis of Sympathetic Activation During Sleep

Studies that research the complex neurobiological mechanisms linking sympathetic nervous system activation to involuntary body and limb movements during sleep have demonstrated that autonomic regulation is integrated and orchestrated by key brain-stem structures. Zhang et al. investigated the function of neurons within the rVMM in mice, using optogenetic stimulation, discovering that excitatory rVMM neurons simultaneously increased both muscle tone and sympathetic output, while inhibitory neurons reduced both [10]. These neurons projected to motor circuits as well as sympathetic preganglionic neurons, positioning them as dual regulators of both movement and autonomic tone. Notably, when excitatory neurons were inhibited, the mice showed impaired locomotion and diminished sympathetic tone; when inhibitory neurons were silenced, REM sleep atonia was abolished, suggesting a failure of the parasympathetic system to assert dominance during REM, revealing a shared central mechanism through which body movements and sympathetic surges during sleep may arise, providing a potential explanation for phenomena like periodic limb movements or REM behavior disorder [10].

In another study, Szymusiak et al. highlighted the thermoregulatory role of the hypothalamus, specifically, the preoptic/anterior hypothalamic area, as a key integrator of sleep and autonomic processes [11]. The authors explained how core body temperature declines during sleep, driven by reduced sympathetic tone and metabolic output. Simultaneously, distal skin temperature increases, indicating peripheral vasodilation and autonomic quiescence, especially at sleep onset [11]. Intriguingly, subsets of warm-sensing neurons within the preoptic area become spontaneously active during NREM sleep, promoting parasympathetic dominance and possibly suppressing motor activity [11]. Since these thermal shifts vary by sleep stage and rely on autonomic integrity, disruptions in sympathetic tone, for example, due to stress or sleep disorders, may destabilize thermoregulation and provoke involuntary arousals or movement episodes.

This state-dependence of sympathetic output is further illustrated in the work of Donadio et al., who examined patients with narcolepsy with cataplexy; using direct recordings of MSNA and skin sympathetic activity (SSA), the researchers showed that patients with narcolepsy lack the normal sympathetic activation seen during REM sleep [12]. REM sleep-onset periods (SOREMPs) in these patients more closely resembled NREM sleep in their autonomic profile, lacking the typical sympathetic surges and failing to raise blood pressure or heart rate [12]. Sympathetic activation during sleep, then, is not only stage-specific but also susceptible to pathologic modulation in neurodegenerative or sleep disorders. The absence of sympathetic tone in REM may help explain reduced motor inhibition and the presence of REM sleep behavior disorder features in narcolepsy with cataplexy.

Noradrenergic pathways may represent an underrecognized component of sleep-related movement disorders. The locus coeruleus, the principal source of cerebral norepinephrine, is highly active during wakefulness, decreases firing during non-rapid eye movement sleep, and becomes nearly silent during rapid eye movement sleep [13]. Because norepinephrine strongly modulates arousal threshold, cardiovascular tone, cortical excitability, and spinal motor responsiveness, persistent nocturnal noradrenergic activation could contribute to sleep fragmentation, autonomic surges, and movement instability [14]. This framework may help explain why sympathetic activation frequently accompanies or precedes motor events during sleep. Measuring sympathetic activity in routine practice may be possible by using a combination of non-invasive, indirect hemodynamic markers, autonomic reflex testing, and validated heart rate variability (HRV) analysis [15].

## 3. Sympathetic Activation in Sleep-Related Movement Disorders

Moving from REM-related conditions to sleep-related movement disorder, Jin et al. offered compelling evidence that sympathetic activation may precede or even provoke limb movements in RLS [16]. The authors analyzed sleep data from RLS patients and matched controls to find that RLS patients with moderate to severe RLS experienced frequent spikes in heart rate, a surrogate for sympathetic bursts, even in the absence of leg movements [16]. Moreover, nearly half of the leg movements in RLS patients were accompanied by increases in heart rate, compared to approximately 20% in healthy controls [16]. This temporal relationship suggests that autonomic activation is not merely a response to movement but may also act as a precipitating factor, especially in individuals with heightened RLS severity.

Gupta and Gupta proposed that increased sympathetic tone may underlie elevated leg movement indices in both Periodic Limb Movement Disorder (PLMD) and atopic dermatitis, noting that both conditions, although clinically distinct, share a pattern of nocturnal sympathetic arousal [17]. However, recent evidence shows that sympathetic overactivation is also present, either preceding or co-occurring with leg movements, suggesting an autonomic component independent of dopamine [18]. For instance, Jin et al. found that in moderate-to-severe RLS, sympathetic bursts (measured via heart rate increases) occurred frequently even without limb movements and were more prevalent in patients with higher RLS severity [16]. This strongly suggests that autonomic dysregulation is not just a response to movements but may trigger them. The implication is that sympathetic arousals and dopaminergic dysfunction may be co-conspirators in generating RLS symptoms. Ferrillo et al. investigated cerebral and autonomic activity preceding PLMS in sleep [7]. The study involved five subjects diagnosed with PLMD without RLS. Findings indicated that PLMS onset is preceded by significant increases in heart rate and delta activity power, suggesting a common brainstem system regulating both cardiac and cortical activities about PLMS. Guggisberg et al. investigated the role of the sympathetic nervous system in the pathophysiology of PLMS [19]. The researchers found that sympathetic activation, as measured by heart rate variability spectra, was greater for PLMS than for other types of leg movements during sleep. Additionally, they observed that gamma synchronization in the EEG began 1 to 2 s earlier for isolated leg movements and respiratory-related leg movements than for PLMS [19]. These findings suggest a significant involvement of the sympathetic nervous system in PLMS and indicate that autonomic activations are correlated with electromyographic activity and EEG delta activity. In a recent study by Benbir Senel et al., periodic, isolated, and short-interval leg movements in wakefulness before sleep onset were analyzed in patients with RLS, and the analysis of spectral EEG and heart rate changes demonstrated that prominent cortical and cardiac activation is associated with leg movements in wakefulness [20].

RSD may represent an example of a bridge between theories. The pathophysiology of RSD includes both iron deficiency and increased sympathetic tone as suspected mechanisms [6]. They argue that these children exhibit frequent large movements during sleep with associated daytime symptoms, even in the absence of classic RLS [21]. The authors suggest that instability in arousal regulation, potentially driven by autonomic imbalance, could be a core mechanism [6]. Here, the intersection between neurochemical (iron/dopamine) and autonomic (sympathetic) pathways is most evident.

Although dopamine plays a major role in the pathophysiology of sleep-related movement disorders, its effects are mostly indirect, while substantial evidence accumulates on the effects of noradrenaline (NA). There is direct evidence supporting the role of noradrenaline on sleep/REM sleep regulation [22], which is further supported by the increased level of noradrenaline upon sleep/REM sleep loss [23,24]. Malik and colleagues proposed that sustained elevation of noradrenaline following REM sleep deprivation increases neuronal and brain excitability, primarily through the activation of α1- -adrenoceptor mediated mechanisms with associated modulation of glutamatergic and GABAergic neurotransmission [25]. Acute and chronic noradrenergic exposure have also been shown to differentially affect cortical excitability through modulation of glutamatergic facilitation and GABAergic inhibition [26]. In addition, NA has also been shown to act as an antioxidant by chelating ferrous iron (Fe^2+^); while this chelation is protective at lower concentrations, higher concentrations of NA also result in increased lipid peroxidation and neurotoxicity [27].

In sleep-related movement disorders, as in RLS, PLMS and RSD, there is an increased sympathetic activity and elevated noradrenaline release, which constitute a primary link to increased cardiovascular risk [28]. This heightened sympathetic nervous system activity leads to a state of autonomic dysfunction characterized by nocturnal hypertension, tachycardia, and a reduced dipping of blood pressure, which collectively raise the risk of cardiovascular diseases in sleep-related movement disorders.

## 4. Diagnostic Assessment of Autonomic Dysfunction

The diagnosis of sleep-related movement disorders increasingly relies on the measurement of sympathetic nervous system activity during sleep. DelRosso et al. described the clinical entity of RSD, characterized by large body movements during sleep accompanied by daytime dysfunction [6]. In their work, they emphasized that while the exact pathophysiology of RSD remains unclear, sympathetic overactivity and iron deficiency are suspected to play contributory roles. The diagnosis requires polysomnographic evidence of more than five large movements per hour. The authors suggest that the observed movement may arise from instability in sleep regulation associated with autonomic imbalance [6]. Similarly, research by Chiang et al. used HRV to assess sympathetic activity during the first hour of sleep in patients with OSA [29]. Distinguishing between sympathetic nervous system (SNS) activation that is primary (due to chronic hyperarousal) versus secondary (due to sleep fragmentation itself) involves analyzing the temporal relationship between arousals, blood oxygen levels, and heart rate variability (HRV) during sleep, typically via PSG. Direct assessment of sympathetic nerve activity (MSNA) shows that sleep fragmentation (secondary) leads to massive, transient surges upon arousal, whereas primary hyperarousal (primary) causes sustained high activity throughout the night. A high low-frequency/high-frequency (LF/HF) ratio during NREM sleep indicates sympathetic dominance, typical of chronic secondary fragmentation, which may also help in differentiation. Their findings revealed that while parasympathetic tone typically increases over this period (as indicated by rising RMSSD values), snoring events and delayed sleep onset were associated with spikes in the LF/HF ratio, a marker of sympathetic dominance [29]. Notably, patients with severe OSA exhibited a much higher sympathetic response to snoring. These data highlight how HRV metrics during sleep onset can help identify periods of autonomic stress, which may be implicated in triggering leg or body movements.

The utility of SKNA was further supported by He et al., who measured sympathetic bursts in OSA patients using a technique called neuECG [30]. They found significantly higher SKNA activity in OSA patients compared to controls, especially in association with arousals. Significantly, SKNA bursts corresponded with both NREM-to-REM transitions and movement episodes, suggesting that this non-invasive technique could serve as a biomarker for sympathetic-linked movement phenomena during sleep. Similarly, measuring SSA via sympathetic sudomotor responses is a practical tool in the evaluation of both central and peripheral autonomic dysfunction [31]. Sudomotor response abnormalities from the neck area were demonstrated in OSA, which were reversed after effective treatment [32]. However, a limited number of studies failed to demonstrate significant changes in SSA in patients with RLS compared to control groups [33,34]. In terms of technology, Schnall and colleagues described how Peripheral Arterial Tonometry (PAT) has become a reliable, home-based method to detect sympathetic-mediated vasoconstriction [35]. Since sympathetic tone modulates arterial volume in the distal fingers, PAT has been validated as a proxy for autonomic arousals, often used in portable sleep diagnostics. This method supports both the detection and monitoring of sympathetic activity related to leg movements or awakenings.

In a study by Bertisch et al., beat-by-beat photoplethysmographic arterial pressure and popliteal artery blood flow velocity were measured, in addition to standard EKG, and baroreflex function was assessed [1]. The authors observed reduced cardiovagal baroreflex gain and increased calf vascular resistance in patients with RLS, independent of changes in sleep structure, demonstrating a direct effect of RLS on cardiovascular autonomic control, specific to the arterial baroreflex and vascular resistance.

## 5. Therapeutic Options for Sympathetic Activation During Sleep

From a therapeutic standpoint, most current treatments target either the underlying triggers of sympathetic activation or the resulting movement disturbances. In RSD, iron supplementation is the only treatment studied to date [36]. For patients with OSA, treatment with Continuous Positive Airway Pressure (CPAP) has been shown to attenuate sympathetic surges, especially during REM sleep, a stage known for cardiovascular instability [37]. In their review, Alzoubaidi and Mokhlesi outlined how REM-related OSA is associated with disproportionately high sympathetic activation, and that even when apnea-hypopnea indices are normal during non-REM, untreated REM OSA can still lead to hypertension and insulin resistance [38]. Therefore, treating REM OSA with sufficient CPAP coverage is critical to controlling sympathetic overload and possibly reducing movement-related arousals.

In patients with RLS and PLMD, treatments such as alpha-2-delta ligands or iron therapy may also attenuate the frequency and intensity of sympathetic surges. However, this is an area requiring further study. Significantly, as shown by Jin et al., only moderate to severe RLS was associated with abnormal sympathetic patterns, suggesting that treatment may be especially crucial in patients above a certain severity threshold [16]. GABA supplementation may also support parasympathetic tone and reduce sympathetic dominance. A study by Guimarães et al. showed that sedentary overweight women receiving GABA supplementation showed improvements in sleep efficiency, HRV, and depression scores [39].

Although the use of dopaminergic agonists is limited due to the risk of augmentation in only a small percentage of patients in whom other therapeutic options have contraindications or side effects, it was demonstrated that the increased PLMS-related HR response and elevated HRV were normalized in patients with RLS [40].

Lastly, non-pharmacological approaches such as gentle exercise programs may offer an effective intervention not only for RLS but also for improving sleep, mood, and blood pressure [41].

## 6. Integrated Pathophysiological Model

Emerging evidence suggests that the pathophysiology of sleep-related movement disorders may result from an interplay between iron deficiency, dopamine dysfunction, and sympathetic activation [42]. RLS is an example of this complex relationship. Iron is a critical cofactor for dopamine synthesis, and its deficiency can impair dopaminergic neurotransmission, leading to the hallmark sensory and motor symptoms of RLS. Additionally, iron deficiency may contribute to increased sympathetic nervous system activity, which has been observed in RLS patients [16]. This heightened sympathetic tone can exacerbate sleep disturbances and may further disrupt dopaminergic pathways, creating a vicious cycle that amplifies RLS symptoms [18]. Understanding these interconnected mechanisms is crucial for developing comprehensive treatment strategies that address both the neurological and autonomic components of RLS.

**Figure 1:**
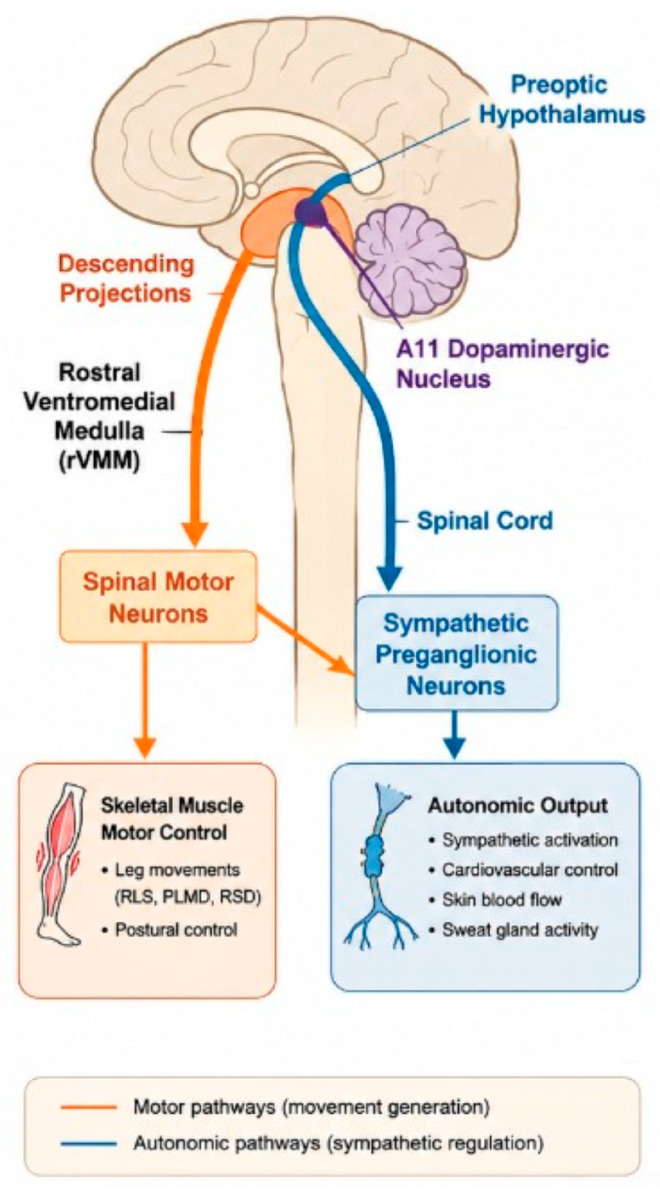
Shared pathways of motor and autonomic control. At the center of this integration is the rostral ventromedial medulla, with descending projections that simultaneously target spinal motor neurons and sympathetic preganglionic neurons. This dual innervation enables the rVMM to regulate both voluntary and involuntary body movements as well as autonomic tone. The preoptic hypothalamus, known for its role in sleep initiation and thermoregulation, also contributes to autonomic modulation, particularly in adjusting sympathetic output during transitions between sleep stages. Meanwhile, the A11 dopaminergic nucleus, the primary source of spinal dopamine, projects to similar regions and modulates spinal motor excitability. When dopamine transmission is impaired, such as in conditions of iron deficiency, these pathways may become dysregulated, leading to heightened motor activity and sympathetic surges. Together, these regions form a functional network that governs both motor execution and autonomic balance. Disruptions in any part of this circuit, whether through dopaminergic dysfunction, iron deficiency, or stress-induced sympathetic hyperactivity, can result in the co-occurrence of movement-related arousals and sleep fragmentation, characteristic of disorders like RLS, PLMD, and RSD. Orange arrows indicate motor pathways, blue arrows indicate autonomic/sympathetic pathways, black labels denote neuroanatomical structures, and purple labeling identifies the A11 dopaminergic dopaminergic system.

The classic understanding of RLS and PLMD centers on dopaminergic dysfunction and iron deficiency. Iron is a critical cofactor for tyrosine hydroxylase, the rate-limiting enzyme in dopamine synthesis. Therefore, iron deficiency impairs dopaminergic neurotransmission, particularly in subcortical areas, such as the A11 dopaminergic nucleus, which projects to spinal motor circuits [43]. Figure 1 illustrates the shared neuroanatomical pathways involved in both motor and autonomic control, emphasizing how overlapping brain structures influence sleep-related movement and arousal regulation.

Another possible theory explaining a potential overlap in sympathetic activation and dopamine dysfunction is to find a shared anatomical substrate, potentially the rVMM and the Hypothalamus. As mentioned above, Zhang et al. described a population of neurons in the rVMM that project to both sympathetic preganglionic neurons and somatomotor circuits [10]. There may be a shared neuroanatomical basis for motor activity and autonomic tone, suggesting a natural interface for the dopaminergic–autonomic convergence in movement disorders.

**Figure 2:**
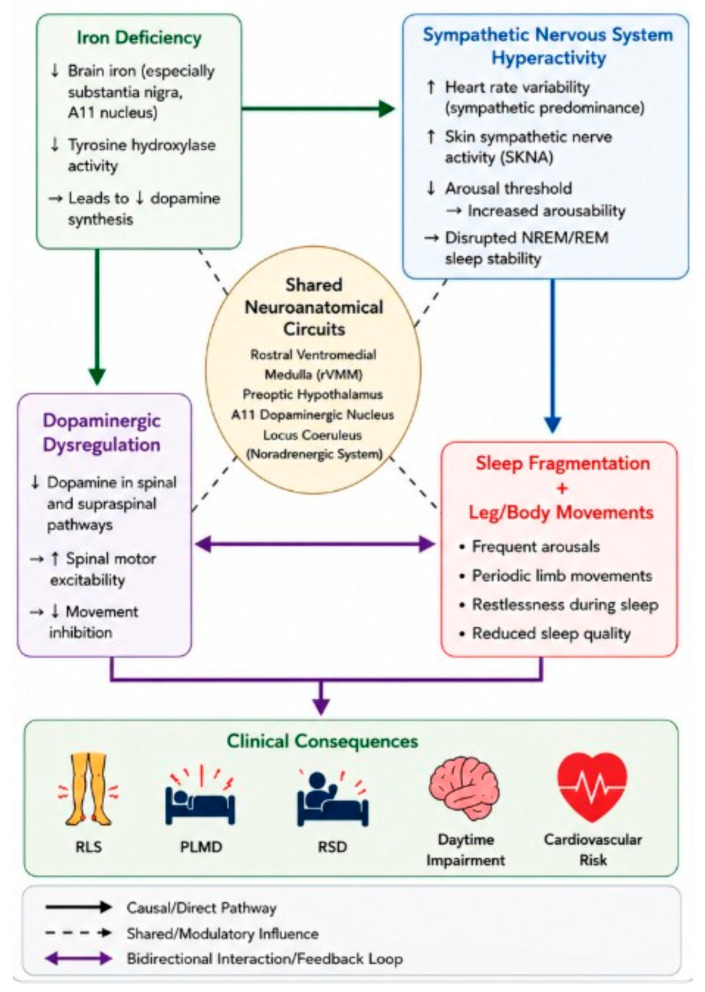
Proposed mechanistic interactions among iron deficiency, dopaminergic dysfunction, sympathetic nervous system hyperactivity, and sleep fragmentation in restless sleep–related disorders, contributing to RLS, PLMD, RSD, daytime impairment, and cardiovascular risk. Solid arrows indicate direct or causal pathways (e.g., iron deficiency leading to reduced dopamine synthesis or sympathetic hyperactivity contributing to sleep fragmentation). Dashed arrows indicate shared or modulatory influences through common neuroanatomical circuits. Purple bidirectional arrows indicate reciprocal interactions or feedback loops between physiologic processes.

Figure 2 presents an integrated model explaining how iron deficiency, dopaminergic dysregulation, and sympathetic nervous system hyperactivity converge to drive sleep-related movement disorders. The sympathetic hyperactivity (stress-induced overactivity) may also cause or worsen iron deficiency, as the relationship is strongly bidirectional. Chronic stress and sympathetic arousal initiate a cascade of inflammatory and neuroendocrine responses that disrupt how the body absorbs, stores, and uses iron, and resulting in functional iron deficiency via hepcidin upregulation, reduced ferroportin, increased tissue iron sequestration, and driving body into a catabolic state [44]. The cascade begins with iron deficiency, particularly in brain regions such as the substantia nigra and A11 nucleus, which leads to reduced tyrosine hydroxylase activity, a key enzyme in dopamine synthesis [45].

A complementary adrenergic perspective may further strengthen the proposed synergistic model. Iron deficiency and dopaminergic dysfunction have traditionally been emphasized in sleep-related movement disorders, yet both systems interact closely with central noradrenergic networks. Experimental evidence suggests that norepinephrine influences spinal sensorimotor excitability, vigilance, and autonomic outflow, while excessive locus coeruleus activity may promote hyperarousal and sleep fragmentation [13]. In this context, iron deficiency could impair catecholaminergic balance broadly, not only dopamine synthesis, thereby favoring a state of heightened sympathetic tone. The coexistence of reduced dopaminergic inhibition and increased noradrenergic activation may create a permissive environment for abnormal movements, recurrent arousals, and cardiovascular activation during sleep [46]. This model provides a unified pathophysiological framework for disorders like RLS, PLMD, and RSD.

Sympathetic activation may also differ between idiopathic (primary) and secondary RLS, with secondary RLS often linked to higher systemic sympathetic overactivity due to the underlying conditions themselves (e.g., renal failure, iron deficiency), whereas idiopathic RLS is more strongly tied to dopaminergic dysfunction and nocturnal PLMS [47]. While RLS generally involves increased sympathetic activity, particularly during sleep (linked to PLMS), idiopathic RLS is characterized more by central nervous system dopaminergic dysfunction and increased sympathetic activity (as measured by heart rate variability) [48]. In patients with end-stage renal disease (ESRD) and secondary RLS, a markedly increased sympathetic nerve activity (MSNA) persists due to uremia regardless of the presence of RLS symptoms but is further aggravated by the secondary RLS. The autonomic (sympathetic) impact in pregnancy-related RLS is generally related to the acute, temporary nature of the pregnancy-induced metabolic stress rather than chronic systemic overactivity.

## 7. Conclusions

This manuscript examines the growing evidence that sympathetic nervous system activation plays a crucial role in sleep disorders, particularly sleep-related movement disorders, as well as obstructive sleep apnea. While traditional models have focused on dopaminergic dysfunction and iron deficiency, new research highlights the importance of autonomic dysregulation as a coexisting or even primary mechanism in some cases.

Our review shows that sympathetic surges often precede or accompany leg movements, suggesting a causative rather than reactive role. Neuroanatomical studies identify overlapping control centers in the brainstem and hypothalamus that coordinate both motor activity and autonomic output. Additionally, iron deficiency may contribute to both impaired dopamine production and increased sympathetic tone, supporting a unified pathophysiological model.

The manuscript proposes an integrated framework that links these mechanisms and offers insight into more comprehensive diagnostic and therapeutic approaches for sleep-related movement disorders. Although it largely describes the current advances in pathophysiology of sleep-related movement disorders, mainly RLS, PLMD and RSD, as it should be interpreted in light of some limitations. Box 1 summarizes key findings linking sympathetic activation, dopaminergic dysfunction, and iron deficiency, and outlines their implications for integrated diagnostic and therapeutic approaches. The study design introduces certain methodological constraints. For example, the reliance on cross-sectional data, self-reported measures or observational methods may increase the risk of desirability bias, or unmeasured confounding variables. Future studies with larger and more diverse populations should incorporate a broader range of variables and consider alternative theoretical frameworks to provide a more comprehensive understanding of the phenomenon. Furthermore, exploring potential moderating and mediating variables could help uncover the underlying mechanisms of sympathetic activation and sleep-related movements.

Box 1 **Clinical Summary of Autonomic Dysfunction in Sleep-Related Movement Disorders**

**Clinical Summary Box**

**Background**

Sleep-related movement disorders—including Restless Legs Syndrome (RLS), Periodic Limb Movement Disorder (PLMD), and Restless Sleep Disorder (RSD)—have traditionally been linked to dopaminergic dysfunction and iron deficiency. Emerging evidence highlights a contributory role of autonomic nervous system dysregulation.

**Key Findings**

- Sympathetic nervous system activation frequently precedes or accompanies motor events, suggesting a potential causal role.
- Electrophysiological and heart rate variability studies demonstrate sympathetic bursts associated with limb movements and arousals.
- Iron deficiency exacerbates both dopaminergic dysfunction and autonomic imbalance, supporting a unified pathophysiological model.

**Pathophysiological Model**

A multidimensional framework integrating:

- Dopaminergic dysfunction;
- Iron metabolism abnormalities;
- Sympathetic (autonomic) activation.

**Clinical Implications**

- Reconsider sleep-related movement disorders as involving autonomic dysfunction in addition to classical mechanisms.
- Broaden management strategies to include:
  o Optimization of continuous positive airway pressure (CPAP) where applicable;
  o Pharmacologic modulation of sympathetic activity;
  o Behavioral interventions targeting autonomic regulation.

**Conclusions**

Sympathetic activation may function as a primary trigger of sleep-related motor events rather than a secondary response, supporting a shift toward integrated diagnostic and therapeutic approaches.

## Data Availability

There is no data to share because this is a review and not an original research.

## Abbreviations

The following abbreviations are used in this manuscript:

RLS	Restless Legs Syndrome
PLMD	Periodic Limb Movement Disorder
PLMS	Periodic Limb Movements of Sleep
RSD	Restless Sleep Disorder
OSA	Obstructive Sleep Apnea
HRV	Heart Rate Variability
SKNA	Skin Sympathetic Nerve Activity
MSNA	Muscle Sympathetic Nerve Activity
SSA	Skin Sympathetic Activity
PAT	Peripheral Arterial Tonometry
PSG	Polysomnography
ECG	Electrocardiogram
REM	Rapid Eye Movement
NREM	Non-Rapid Eye Movement
rVMM	Rostral Ventromedial Medulla
A11	A11 Dopaminergic Nucleus
CPAP	Continuous Positive Airway Pressure
GABA	Gamma-Aminobutyric Acid

## References

[1] Bertisch S.M., Muresan C., Schoerning L., Winkelman J.W., Taylor J.A. (2016). Impact of Restless Legs Syndrome on Cardiovascular Autonomic Control. Sleep.

[2] Figorilli M., Puligheddu M., Congiu P., Ferri R. (2017). The Clinical Importance of Periodic Leg Movements in Sleep. Curr. Treat. Options Neurol..

[3] Mansukhani M.P., Covassin N., Somers V.K. (2019). Neurological Sleep Disorders and Blood Pressure: Current Evidence. Hypertension.

[4] Sarode R., Nikam P.P. (2023). The Impact of Sleep Disorders on Cardiovascular Health: Mechanisms and Interventions. Cureus.

[5] Guo S., Huang J., Jiang H., Han C., Li J., Xu X., Zhang G., Lin Z., Xiong N., Wang T. (2017). Restless Legs Syndrome: From Pathophysiology to Clinical Diagnosis and Management. Front. Aging Neurosci..

[6] DelRosso L.M., Bruni O., Ferri R. (2020). Heart rate variability during sleep in children and adolescents with restless sleep disorder: A comparison with restless legs syndrome and normal controls. J. Clin. Sleep Med..

[7] Ferrillo F., Beelke M., Canovaro P., Watanabe T., Aricò D., Rizzo P., Garbarino S., Nobili L., De Carli F. (2004). Changes in cerebral and autonomic activity heralding periodic limb movements in sleep. Sleep Med..

[8] Kerman I.A. (2008). Organization of brain somatomotor-sympathetic circuits. Exp. Brain Res..

[9] Sforza E., Roche F., Pichot V. (2019). Determinants of Nocturnal Cardiovascular Variability and Heart Rate Arousal Response in Restless Legs Syndrome (RLS)/Periodic Limb Movements (PLMS). J. Clin. Med..

[10] Zhang Z., Su J., Tang J., Chung L., Page J.C., Winter C.C., Liu Y., Kegeles E., Conti S., Zhang Y. (2024). Spinal projecting neurons in rostral ventromedial medulla co-regulate motor and sympathetic tone. Cell.

[11] Szymusiak R. (2018). Body temperature and sleep. Handb. Clin. Neurol..

[12] Donadio V., Liguori R., Vandi S., Giannoccaro M.P., Pizza F., Leta V., Plazzi G. (2014). Sympathetic and cardiovascular changes during sleep in narcolepsy with cataplexy patients. Sleep Med..

[13] Aston-Jones G., Bloom F.E. (1981). Activity of norepinephrine-containing locus coeruleus neurons in behaving rats anticipates fluctuations in the sleep-waking cycle. J. Neurosci..

[14] Bellesi M., Castelnovo A. (2026). Why do we sleepwalk? A noradrenergic hypothesis of NREM sleep parasomnias. Sleep Med. Rev..

[15] Seravalle G., Dimitriadis K., Dell’Oro R., Grassi G. (2013). How to assess sympathetic nervous system activity in clinical practice. Curr. Clin. Pharmacol..

[16] Jin B., Wang A., Earley C., Allen R. (2020). Moderate to severe but not mild RLS is associated with greater sleep-related sympathetic autonomic activation than healthy adults without RLS. Sleep Med..

[17] Gupta M.A., Gupta A.K. (2020). An elevated leg movement index during sleep in atopic dermatitis and periodic leg movement disorder may be an indication of sympathetic activation common to both. J. Clin. Sleep Med..

[18] Bergmann M., Heidbreder A., Stefani A., Raccagni C., Brandauer E., Rudzki D., Fischer M.B., Rossmanith E., Pasztorek M., Löscher W.N. (2021). Signs of sympathetic and endothelial cell activation in the skin of patients with restless legs syndrome. Sleep Med..

[19] Guggisberg A.G., Hess C.W., Mathis J. (2007). The significance of the sympathetic nervous system in the pathophysiology of periodic leg movements in sleep. Sleep.

[20] Benbir Senel G., Tunali A., Demirel O., Köse S., Cakir V., Resadiyeli B., Karadeniz D., Ferri R. (2025). Spectral EEG and heart rate changes associated with leg movements during the suggested immobilization test in patients with restless legs syndrome. J. Sleep Res..

[21] DelRosso L.M., Silvestri R., Ferri R. (2021). Restless Sleep Disorder. Sleep Med. Clin..

[22] Gottesmann C. (2011). The involvement of noradrenaline in rapid eye movement sleep mentation. Front. Neurol..

[23] Giri S., Mehta R., Mallick B.N. (2023). REM Sleep Loss-Induced Elevated Noradrenaline Plays a Significant Role in Neurodegeneration: Synthesis of Findings to Propose a Possible Mechanism of Action from Molecule to Patho-Physiological Changes. Brain Sci..

[24] Mehta R., Singh S., Khanday M.A., Mallick B.N. (2017). Reciprocal changes in noradrenaline and GABA levels in discrete brain regions upon rapid eye movement sleep deprivation in rats. Neurochem. Int..

[25] Mallick B.N., Singh A. (2011). REM sleep loss increases brain excitability: Role of noradrenalin and its mechanism of action. Sleep Med. Rev..

[26] Kuo H.I., Paulus W., Batsikadze G., Jamil A., Kuo M.F., Nitsche M.A. (2017). Acute and Chronic Noradrenergic Effects on Cortical Excitability in Healthy Humans. Int. J. Neuropsychopharmacol..

[27] Singh A., Das G., Kaur M., Mallick B.N. (2019). Noradrenaline Acting on Alpha1 Adrenoceptor as well as by Chelating Iron Reduces Oxidative Burden on the Brain: Implications with Rapid Eye Movement Sleep. Front. Mol. Neurosci..

[28] Katsi V., Katsimichas T., Kallistratos M.S., Tsekoura D., Makris T., Manolis A.J., Tousoulis D., Stefanadis C., Kallikazaros I. (2014). The association of Restless Legs Syndrome with hypertension and cardiovascular disease. Med. Sci. Monit..

[29] Chiang J.K., Lin Y.C., Kao Y.H. (2023). Oscillation of Sympathetic Activity in Patients with Obstructive Sleep Apnea during the First Hour of Sleep. Healthcare.

[30] He W., Tang Y., Meng G., Wang D., Wong J., Mitscher G.A., Adams D., Everett T.H., Chen P.S., Manchanda S. (2020). Skin sympathetic nerve activity in patients with obstructive sleep apnea. Heart Rhythm..

[31] Schondorf R. (1993). New investigations of autonomic nervous system function. J. Clin. Neurophysiol..

[32] Korkmaz B., Benbir Senel G., Kiziltan M.E., Karadeniz D. (2016). Demonstration of sympathetic dysfunction in patients with obstructive sleep apnea syndrome by measuring sympathetic skin responses from the neck. Sleep Med..

[33] Erdal Y., Akdogan O., Nalbantoglu M., Kavasoglu G., Emre U. (2020). Autonomic dysfunction in restless legs syndrome. Sleep Breath..

[34] Shukla G., Gupta A., Pandey R.M., Kalaivani M., Goyal V., Srivastava A., Behari M. (2014). What features differentiate unilateral from bilateral restless legs syndrome? A comparative observational study of 195 patients. Sleep Med..

[35] Schnall R.P., Sheffy J.K., Penzel T. (2022). Peripheral arterial tonometry-PAT technology. Sleep Med. Rev..

[36] DelRosso L.M., Picchietti D.L., Ferri R. (2021). Comparison between oral ferrous sulfate and intravenous ferric carboxymaltose in children with restless sleep disorder. Sleep.

[37] Yu L., Shu L., Gao S., Li L. (2025). CPAP improves sleep stability and attenuates acute nocturnal hypertension (NBPF) in OSA, with maximal benefits in severe cases. Front. Neurol..

[38] Alzoubaidi M., Mokhlesi B. (2016). Obstructive sleep apnea during rapid eye movement sleep: Clinical relevance and therapeutic implications. Curr. Opin. Pulm. Med..

[39] Guimarães A.P., Seidel H., Pires L.V.M., Trindade C.O., Baleeiro R.d.S., de Souza P.M., e Silva F.G.D., Coelho D.B., Becker L.K., de Oliveira E.C. (2024). GABA Supplementation, Increased Heart-Rate Variability, Emotional Response, Sleep Efficiency and Reduced Depression in Sedentary Overweight Women Undergoing Physical Exercise: Placebo-Controlled, Randomized Clinical Trial. J. Diet. Suppl..

[40] Manconi M., Ferri R., Zucconi M., Clemens S., Rundo F., Oldani A., Ferini-Strambi L. (2011). Effects of acute dopamine-agonist treatment in restless legs syndrome on heart rate variability during sleep. Sleep Med..

[41] Innes K.E., Selfe T.K. (2012). The Effects of a Gentle Yoga Program on Sleep, Mood, and Blood Pressure in Older Women with Restless Legs Syndrome (RLS): A Preliminary Randomized Controlled Trial. Evid. Based Complement. Altern. Med..

[42] Alzaabi F.M., Al Tarawneh D.J., Al Tarawneh Y.J., Khan A., Khan M.A.M., Siddiqui T.W., Siddiqui R.W., Nishat S.M.H., Alzaabi A.A., Siddiqui S.W. (2025). Restless Legs and Iron Deficiency: Unraveling the Hidden Link and Unlocking Relief. Cureus.

[43] Allen R. (2004). Dopamine and iron in the pathophysiology of restless legs syndrome (RLS). Sleep Med..

[44] Reid B.M., Georgieff M.K. (2023). The Interaction between Psychological Stress and Iron Status on Early-Life Neurodevelopmental Outcomes. Nutrients.

[45] Dong K., Liu B., Cheng G., Li Y., Xie F., Zhang J., Qian L. (2025). Stress-Induced Dysregulation of Brain Iron Metabolism and Its Links to Neurological Disorders. Biology.

[46] Lüthi A., Nedergaard M. (2025). Anything but small: Microarousals stand at the crossroad between noradrenaline signaling and key sleep functions. Neuron.

[47] Grassi G., Biffi A., Seravalle G., Bertoli S., Airoldi F., Corrao G., Pisano A., Mallamaci F., Mancia G., Zoccali C. (2021). Sympathetic nerve traffic overactivity in chronic kidney disease: A systematic review and meta-analysis. J. Hypertens..

[48] Barone D.A., Ebben M.R., DeGrazia M., Mortara D., Krieger A.C. (2017). Heart rate variability in restless legs syndrome and periodic limb movements of Sleep. Sleep Sci..

